# Prevalence of vitamin D supplement use in Australian residential aged care facilities in November 2014

**DOI:** 10.1186/s13104-017-2721-7

**Published:** 2017-08-10

**Authors:** Pippy Walker, Amanda Miller Amberber, Susan Kurrle, Annette Kifley, Ian D. Cameron

**Affiliations:** 1 John Walsh Centre for Rehabilitation Research, University of Sydney, Kolling Institute of Medical Research, Royal North Shore Hospital, St Leonards, NSW 2065 Australia; 2 NHMRC Cognitive Decline Partnership Centre, Hornsby Ku-ring-gai Health Service, Hornsby, NSW 2077 Australia

**Keywords:** Falls, Frail older adults, Preventive medicine, Residential facilities, Vitamin D

## Abstract

**Objective:**

We sought to establish the prevalence and predictors of adequate vitamin D supplement use, as per current falls prevention guidelines in Australian aged care homes. De-identified medication chart data from November 2014 were collected from pharmacists. The proportion of residents prescribed vitamin D and associations between adequate vitamin D supplementation and state, calcium use and osteoporosis medication use were assessed.

**Results:**

The prevalence of adequate vitamin D supplement use (≥800 IU) was 47.1% of residents (95% CI 41.4, 52.8%). There was no significant difference between states (p = 0.3), however there was large variation between individual facilities (15.9–85.0%). Residents were more likely to be prescribed an adequate dose of vitamin D if they were prescribed a calcium supplement (p = 0.0001) or an osteoporosis medication (p = 0.03).

## Introduction

Falls are a significant cause of harm and hospitalisation in residential aged care [1, 2]. There is strong evidence that vitamin D supplementation is effective in reducing the rate of falls in aged care homes [3]. Although more recent meta-analyses have not shown a significant effect of vitamin D on falls, these reviews include studies conducted outside of the aged care setting and only report on the risk, not rate of falls [4, 5]. Although the Cochrane review did not find a significant effect on the reduction in the risk of falling either, it should be noted that the two largest trials included in this analysis either excluded residents with a low serum 25(OH)D level (579 residents excluded with 25(OH)D <25 nmol/L) or reported a higher median level in a 1% sample of participants (median 47 nmol/L) compared to other studies [3, 6, 7]. Given a recent meta-analysis has found fallers have lower 25(OH)D levels compared to non-fallers, the inclusion of these studies may be masking the true effect as they do not represent the aged care population with widespread deficiency [8].

Nonetheless given with each fall there is increased risk of mortality and morbidity, knowing that vitamin D can reduce the rate of falls in the residential aged care setting provides sufficient reason for its recommendation. This intervention is also supported by the fact that vitamin D supplementation can improve serum 25(OH)D concentrations [9], and it is cost effective [10]. Current best practice guidelines for falls prevention in Australian aged care homes recommend that vitamin D supplements be considered for residents [11]. Although there is no recommended daily intake (RDI) for vitamin D, current guidelines recommend a supplement of at least 800 IU/day, since this has been the minimum daily dosage used in trials that have shown benefit [3, 11].

Despite the current recommendation for vitamin D supplementation in Australian aged care facilities, the uptake of this evidence into practice has not been widely investigated. Of seven identified publications reporting baseline vitamin D supplement use, each has only investigated one aged care facility or one Australian town or region [12–18]. Four of the seven audits were small with less than 100 residents, and the largest study (5391 residents) is now over a decade old [14]. There has been one recent audit in metropolitan WA, reporting a mean prevalence of 41.5% of residents (n = 779), however like most other audits it is not clear whether the adequacy of the dosage prescribed was considered [17]. Of the only two studies to report on dosages, one included residents that were prescribed between 10 and 1000 IU/day and the other only included residents from one state [16, 18]. To date there has been no large scale, multi-state audit of adequate (≥800 IU/day) vitamin D supplement use in Australian aged care homes.

The aim of this study was to establish the prevalence and predictors of adequate (≥800 IU/day) vitamin D supplement use as per current guidelines in Australian aged care homes. The audit served as a pilot to the vitamin D implementation study (ViDAus) focused on addressing the barriers to implementation of this evidence into practice (ANZCTR ID: ACTRN12616000782437).

## Main text

### Methods

Resident medication charts were reviewed from a nominated week in November 2014 by pharmacists that service participating residential aged care facilities in New South Wales, South Australia and Western Australia. Nominated contacts at participating not-for-profit aged care organisations were responsible for approaching pharmacies that supplied packed medications to introduce the study and collect data to ensure information was de-identified, before passing on for analysis. Variables collected included gender, age, whether a vitamin D supplement was prescribed and the daily equivalent dose, whether a calcium supplement was prescribed and whether an osteoporosis medication was prescribed. An Excel spreadsheet including these variables was provided for pharmacies to complete for all residents occupying a facility bed during the nominated week.

The overall proportion of residents receiving adequate vitamin D supplementation and the mean vitamin D dosage among recipients, were calculated with 95% confidence intervals after accounting for clustering by facility using equal weighting for each facility. Prevalence of adequate vitamin D supplementation was also calculated by state and by a de-identified facility number. Associations between adequate vitamin D supplementation and state, calcium use and osteoporosis medication use were assessed after accounting for clustering using the Rao–Scott Chi squared test. All analyses were conducted using Statistical Analysis Software (SAS) version 9.4.

### Results

Of 30 invited facilities (2171 residents), data were received for 21 facilities (1592 residents). There was a reasonable spread of facility size with six facilities having between 20 and 50 residents, nine facilities having 50–99 residents and six facilities with between 100 and 155 residents. Gender and age variables were provided for 1128 residents (69.4% women, mean age 82.4 years). The prevalence of adequate vitamin D supplement use (defined as percentage of residents taking a vitamin D supplement with a mean daily dose of greater than or equal to 800 IU) was 47.1% of residents (95% CI 41.4, 52.8%) as shown in Table 1. 7% (112/1592) of residents were prescribed an inadequate dose of vitamin D (<800 IU/day) and the mean dosage of vitamin D was 1121.6 IU/day (range 200–5000 IU) (95% CI 1044.0, 1199.2 IU). There was no significant difference in the prevalence of vitamin D supplement use between states (p = 0.3), however there was large variation between individual facilities (15.9–85.0%).

**Table 1:** Prevalence and predictors of adequate vitamin D supplement use in Australian aged care facilities in November 2014

	Total cohort (n = 1592)	NSW (n = 366)	SA (n = 566)	WA (n = 660)
Prescribed ≥800 IU/day of vitamin D, n (%)	750 (47.1)	196 (53.6)	246 (43.5)	308 (46.7)
95% CI, %	41.4, 52.8			
Dosage range (IU/day)	800–5000			
p value for association with calcium supplement use^a^	0.0001			
p value for association with osteoporosis medication use^a^	0.03			
p value for comparison between states^a^	0.3			

*CI* confidence interval, *IU* international units

^a^X^2^ p value accounting for clustering

An individual analysis of vitamin D and calcium prescription identified an association between the two. Of all residents that were prescribed a calcium supplement, 63.7% were prescribed an adequate dose (≥800 IU) of vitamin D, compared to only 42.5% of residents not prescribed calcium (p = 0.0001). Similarly an association between the prescription of vitamin D and osteoporosis medications was found. Of residents that were prescribed an osteoporosis medication, 60% were also prescribed vitamin D compared to only 45.8% of residents that were not prescribed an osteoporosis medication (p = 0.03).

### Discussion

This audit of vitamin D supplement use in Australian aged care homes confirms a gap in the translation of evidence into practice. Given previous reports of between 6 and 41.5%, it would seem that vitamin D supplement use has been increasing over the last decade, despite the likelihood of previous reports overestimating the prevalence of adequate vitamin D supplement use since dosage was not considered [12–17].

The variability between individual aged care facilities (15.9–85.0%) indicates both more information is required to interpret this result, and that there should be further investigation into the barriers to the prescription of vitamin D. This may include understanding the mobility level of residents, given the recent release of de-prescribing guidelines highlighting the inappropriateness of vitamin D for residents that are immobile and therefore at very low risk of falls [19].

The variation in dosages prescribed suggests that education on current best practice evidence and guidelines is required across the aged care setting. This is evidenced by the finding that seven per cent of residents were prescribed less than the recommended 800 IU/day. Understanding that calcium supplement and osteoporosis medication use were predictors of the prescription of vitamin D also suggests that targeted education on the prevention and management of falls and osteoporosis is required to help close this evidence to practice gap.

### Conclusions

This research has shown that less than half (47.1%) of the audited aged care residents were prescribed an adequate dose (≥800 IU/day) of vitamin D. Residents that were prescribed calcium or an osteoporosis medication were more likely to be prescribed an adequate dose of vitamin D, suggesting that knowledge of current guidelines may be a barrier to the implementation of this evidence into practice. Given the variation between individual facilities, future research should consider the mobility level of residents to ensure practices are appropriately evaluated. This dose specific audit emphasises the need for a better understanding of the barriers and enablers to implementation, and strategies to increase the use of vitamin D supplements in the aged care setting.

## Limitations

The limitations of this audit relate to data collection methods. Unfortunately the prescription details of equivalent daily dosages were not collected, which would have been useful in light of the recently published literature identifying an increased risk of falls with high monthly dosages of vitamin D [20].

Secondly as some pharmacists do not routinely enter gender and date of birth information into their dispensing software, age and gender variables were not provided for all residents and were therefore not able to be evaluated as potential predictors of vitamin D supplement use.

Thirdly this sample may not be representative of the Australian aged care population, as only three states were included in the sample. A recent audit conducted in Tasmania however has reported similar results with a prevalence of adequate (≥1000 IU/day) vitamin D supplement use of 50% (n = 811) [18].

The final point to note is that just under a third of invited aged care homes were not included in the audit, as not all pharmacies that were contacted provided data. As all data from pharmacies were grouped together by aged care contacts before passing onto the researchers for analysis, it is unclear how many pharmacies contributed, and how many declined since it is possible that pharmacies supply packed medications to more than one facility in an organisation. From the data provided a response rate of 70% (21/30) of facilities for which data was sought and 73.3% of resident beds (1592/2171) within these facilities can be reported.

Whilst the feedback from aged care contacts was that declining pharmacies did not have the time to collate the requested information, for future audits it would be advantageous for researchers to be able to have direct contact with pharmacies to ascertain reasons for declining the invitation to participate and any differences between the two groups of pharmacies to consider the risk of bias. Similarly, it would also be advantageous to have the invited facilities identified to the researchers, to allow for evaluation of whether there is any difference between facilities that did, and did not form part of this audit.

## Abbreviation

ANZCTR ID: Australian New Zealand Clinical Trials Registry Trial Identification
